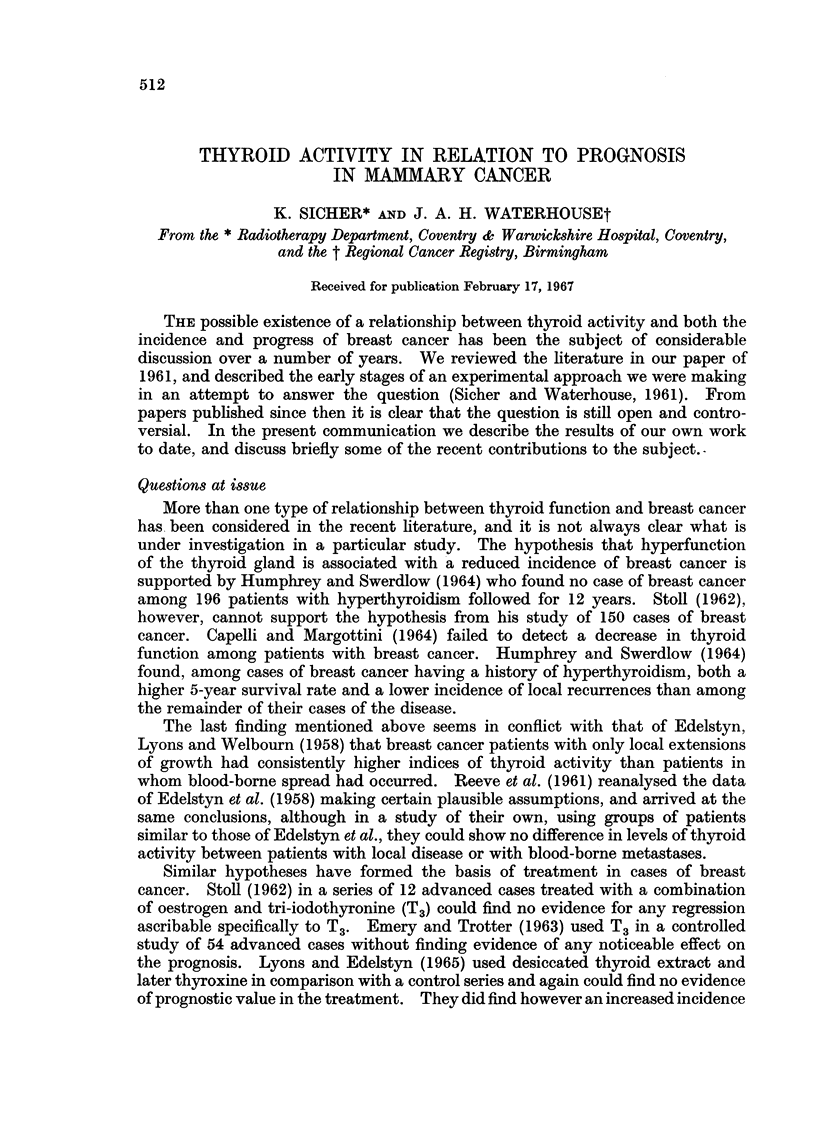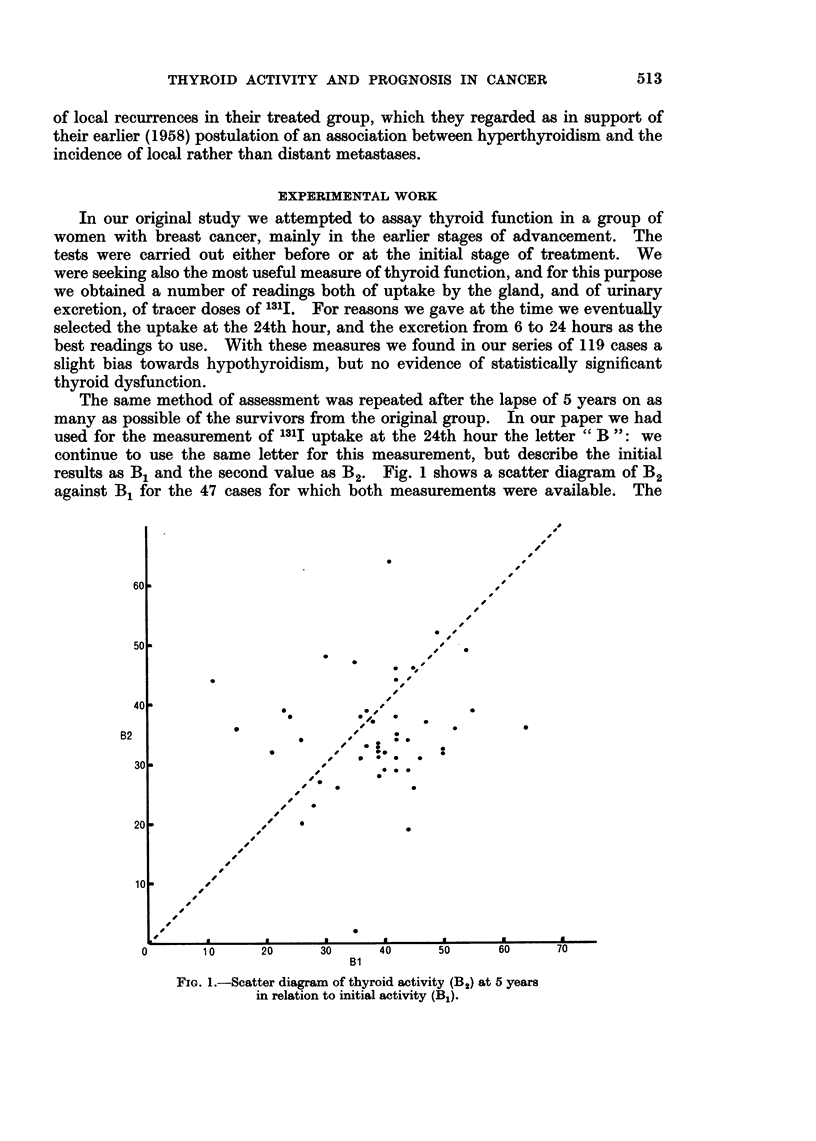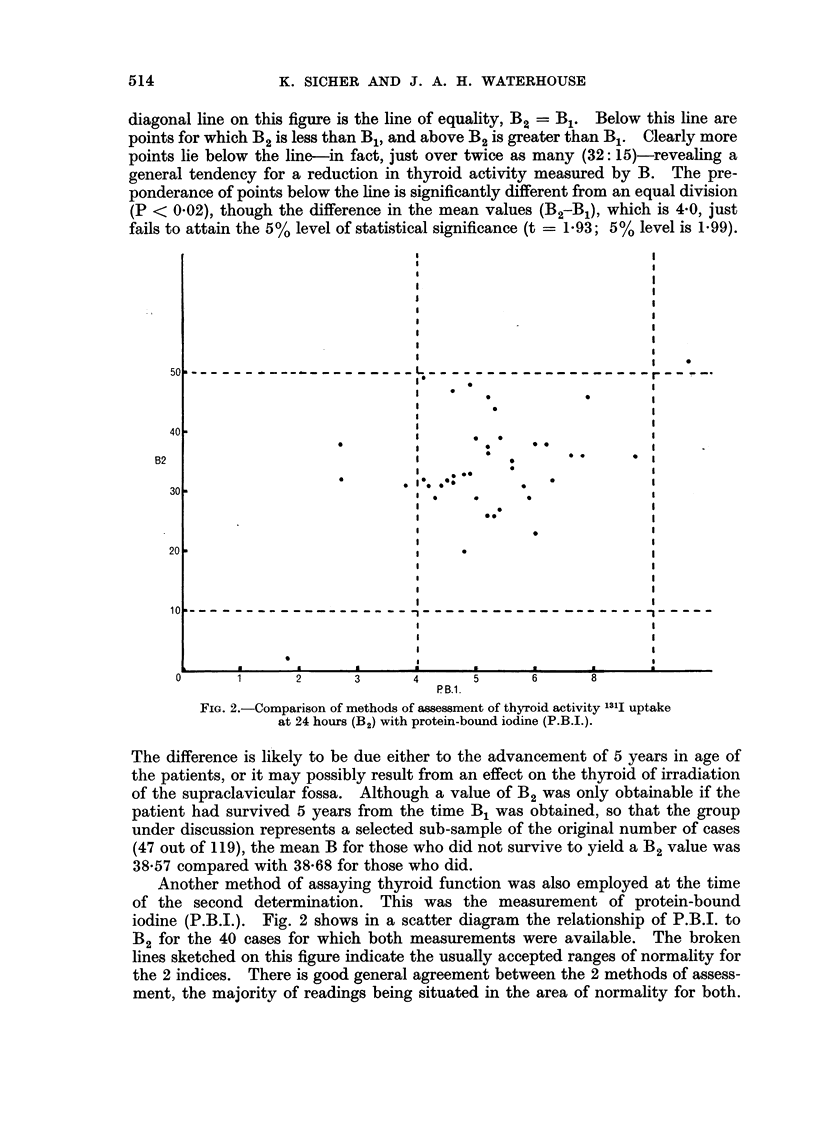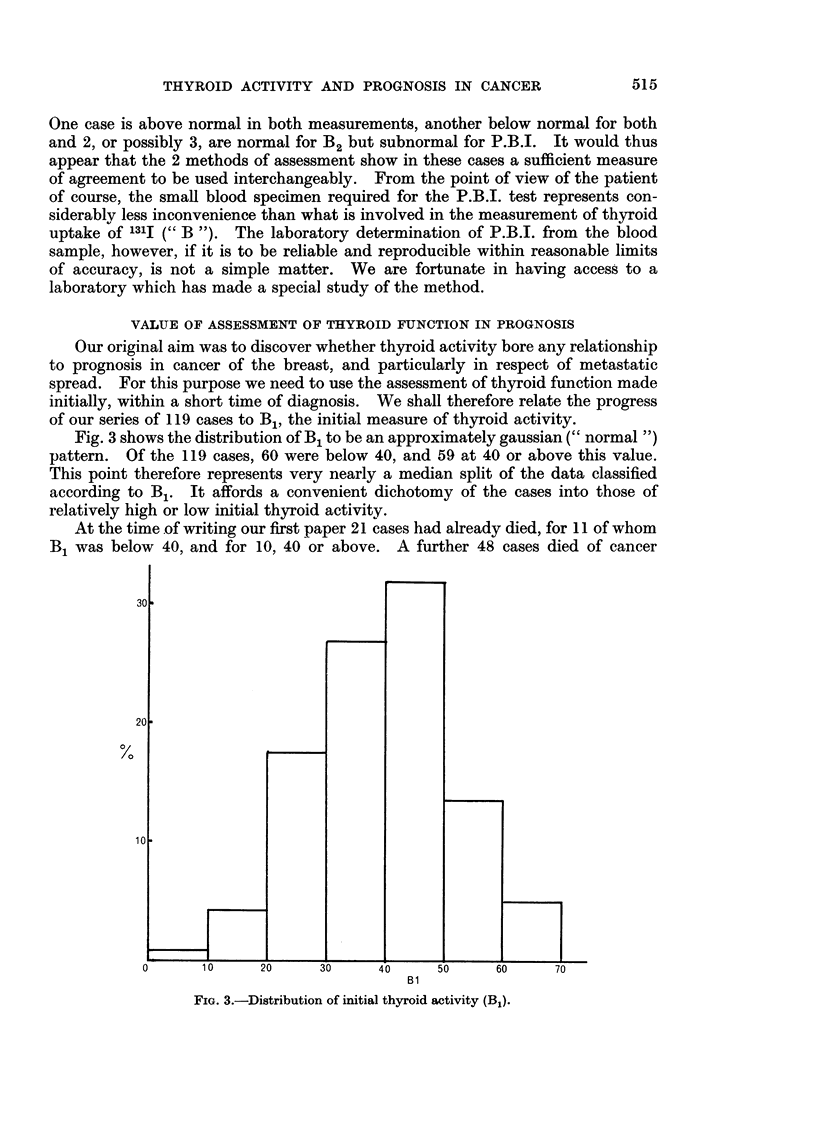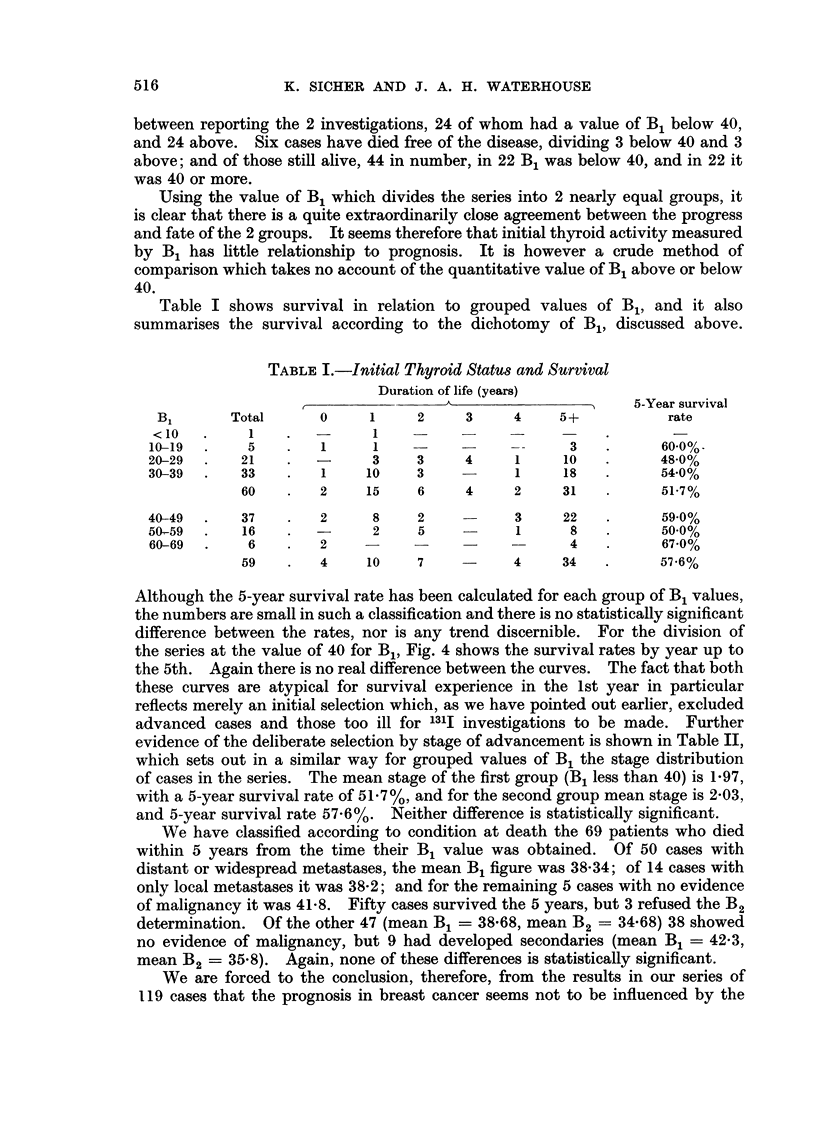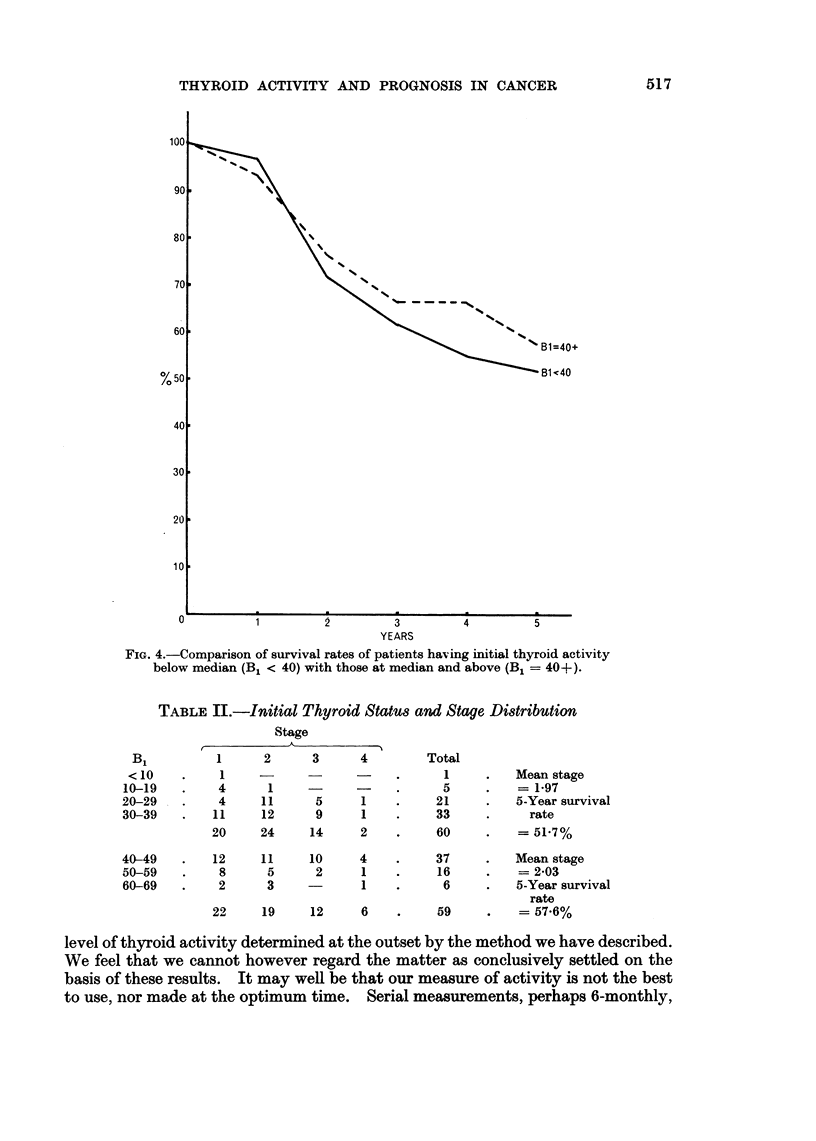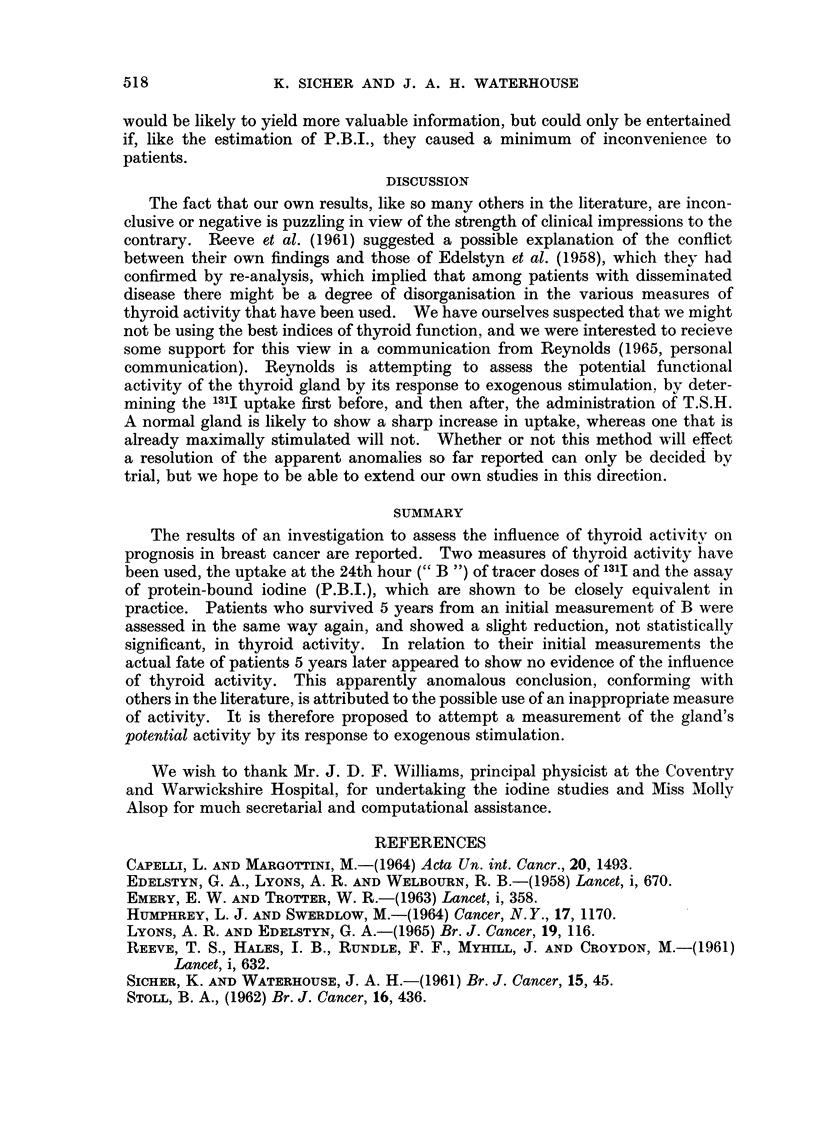# Thyroid activity in relation to prognosis in mammary cancer.

**DOI:** 10.1038/bjc.1967.60

**Published:** 1967-09

**Authors:** K. Sicher, J. A. Waterhouse


### THYROID ACTIVITY IN RELATION TO PROGNOSIS IN MAMMARY CANCER

K. SICHER* AND J. A. H. WATERHOUSE†

*From the * Radiotherapy Department, Coventry & Warwickshire Hospital, Coventry, and the † Regional Cancer Registry, Birmingham*

Received for publication February 17, 1967

THE possible existence of a relationship between thyroid activity and both the incidence and progress of breast cancer has been the subject of considerable discussion over a number of years. We reviewed the literature in our paper of 1961, and described the early stages of an experimental approach we were making in an attempt to answer the question (Sicher and Waterhouse, 1961). From papers published since then it is clear that the question is still open and controversial. In the present communication we describe the results of our own work to date, and discuss briefly some of the recent contributions to the subject.

*Questions at issue*

More than one type of relationship between thyroid function and breast cancer has been considered in the recent literature, and it is not always clear what is under investigation in a particular study. The hypothesis that hyperfunction of the thyroid gland is associated with a reduced incidence of breast cancer is supported by Humphrey and Swerdlow (1964) who found no case of breast cancer among 196 patients with hyperthyroidism followed for 12 years. Stoll (1962), however, cannot support the hypothesis from his study of 150 cases of breast cancer. Capelli and Margottini (1964) failed to detect a decrease in thyroid function among patients with breast cancer. Humphrey and Swerdlow (1964) found, among cases of breast cancer having a history of hyperthyroidism, both a higher 5-year survival rate and a lower incidence of local recurrences than among the remainder of their cases of the disease.

The last finding mentioned above seems in conflict with that of Edelstyn, Lyons and Welbourn (1958) that breast cancer patients with only local extensions of growth had consistently higher indices of thyroid activity than patients in whom blood-borne spread had occurred. Reeve *et al.* (1961) reanalysed the data of Edelstyn *et al.* (1958) making certain plausible assumptions, and arrived at the same conclusions, although in a study of their own, using groups of patients similar to those of Edelstyn *et al.*, they could show no difference in levels of thyroid activity between patients with local disease or with blood-borne metastases.

Similar hypotheses have formed the basis of treatment in cases of breast cancer. Stoll (1962) in a series of 12 advanced cases treated with a combination of oestrogen and tri-iodothyronine ($T_3$) could find no evidence for any regression ascribable specifically to $T_3$. Emery and Trotter (1963) used $T_3$ in a controlled study of 54 advanced cases without finding evidence of any noticeable effect on the prognosis. Lyons and Edelstyn (1965) used desiccated thyroid extract and later thyroxine in comparison with a control series and again could find no evidence of prognostic value in the treatment. They did find however an increased incidence of local recurrences in their treated group, which they regarded as in support of their earlier (1958) postulation of an association between hyperthyroidism and the incidence of local rather than distant metastases.

### EXPERIMENTAL WORK

In our original study we attempted to assay thyroid function in a group of women with breast cancer, mainly in the earlier stages of advancement. The tests were carried out either before or at the initial stage of treatment. We were seeking also the most useful measure of thyroid function, and for this purpose we obtained a number of readings both of uptake by the gland, and of urinary excretion, of tracer doses of $^{131}I$. For reasons we gave at the time we eventually selected the uptake at the 24th hour, and the excretion from 6 to 24 hours as the best readings to use. With these measures we found in our series of 119 cases a slight bias towards hypothyroidism, but no evidence of statistically significant thyroid dysfunction.

The same method of assessment was repeated after the lapse of 5 years on as many as possible of the survivors from the original group. In our paper we had used for the measurement of $^{131}I$ uptake at the 24th hour the letter " B ": we continue to use the same letter for this measurement, but describe the initial results as $B_1$ and the second value as $B_2$. Fig. 1 shows a scatter diagram of $B_2$ against $B_1$ for the 47 cases for which both measurements were available. The

FIG. 1.—Scatter diagram of thyroid activity ($B_2$) at 5 years in relation to initial activity ($B_1$).

diagonal line on this figure is the line of equality, $B_2 = B_1$. Below this line are points for which $B_2$ is less than $B_1$, and above $B_2$ is greater than $B_1$. Clearly more points lie below the line—in fact, just over twice as many (32 : 15)—revealing a general tendency for a reduction in thyroid activity measured by B. The preponderance of points below the line is significantly different from an equal division ($P < 0{\cdot}02$), though the difference in the mean values ($B_2$–$B_1$), which is 4·0, just fails to attain the 5% level of statistical significance ($t = 1{\cdot}93$; 5% level is 1·99).

FIG. 2.—Comparison of methods of assessment of thyroid activity $^{131}I$ uptake at 24 hours ($B_2$) with protein-bound iodine (P.B.I.).

The difference is likely to be due either to the advancement of 5 years in age of the patients, or it may possibly result from an effect on the thyroid of irradiation of the supraclavicular fossa. Although a value of $B_2$ was only obtainable if the patient had survived 5 years from the time $B_1$ was obtained, so that the group under discussion represents a selected sub-sample of the original number of cases (47 out of 119), the mean B for those who did not survive to yield a $B_2$ value was 38·57 compared with 38·68 for those who did.

Another method of assaying thyroid function was also employed at the time of the second determination. This was the measurement of protein-bound iodine (P.B.I.). Fig. 2 shows in a scatter diagram the relationship of P.B.I. to $B_2$ for the 40 cases for which both measurements were available. The broken lines sketched on this figure indicate the usually accepted ranges of normality for the 2 indices. There is good general agreement between the 2 methods of assessment, the majority of readings being situated in the area of normality for both.

One case is above normal in both measurements, another below normal for both and 2, or possibly 3, are normal for $B_2$ but subnormal for P.B.I. It would thus appear that the 2 methods of assessment show in these cases a sufficient measure of agreement to be used interchangeably. From the point of view of the patient of course, the small blood specimen required for the P.B.I. test represents considerably less inconvenience than what is involved in the measurement of thyroid uptake of $^{131}I$ (" B "). The laboratory determination of P.B.I. from the blood sample, however, if it is to be reliable and reproducible within reasonable limits of accuracy, is not a simple matter. We are fortunate in having access to a laboratory which has made a special study of the method.

### VALUE OF ASSESSMENT OF THYROID FUNCTION IN PROGNOSIS

Our original aim was to discover whether thyroid activity bore any relationship to prognosis in cancer of the breast, and particularly in respect of metastatic spread. For this purpose we need to use the assessment of thyroid function made initially, within a short time of diagnosis. We shall therefore relate the progress of our series of 119 cases to $B_1$, the initial measure of thyroid activity.

Fig. 3 shows the distribution of $B_1$ to be an approximately gaussian (" normal ") pattern. Of the 119 cases, 60 were below 40, and 59 at 40 or above this value. This point therefore represents very nearly a median split of the data classified according to $B_1$. It affords a convenient dichotomy of the cases into those of relatively high or low initial thyroid activity.

At the time of writing our first paper 21 cases had already died, for 11 of whom $B_1$ was below 40, and for 10, 40 or above. A further 48 cases died of cancer

FIG. 3.—Distribution of initial thyroid activity ($B_1$).

between reporting the 2 investigations, 24 of whom had a value of $B_1$ below 40, and 24 above. Six cases have died free of the disease, dividing 3 below 40 and 3 above; and of those still alive, 44 in number, in 22 $B_1$ was below 40, and in 22 it was 40 or more.

Using the value of $B_1$ which divides the series into 2 nearly equal groups, it is clear that there is a quite extraordinarily close agreement between the progress and fate of the 2 groups. It seems therefore that initial thyroid activity measured by $B_1$ has little relationship to prognosis. It is however a crude method of comparison which takes no account of the quantitative value of $B_1$ above or below 40.

Table I shows survival in relation to grouped values of $B_1$, and it also summarises the survival according to the dichotomy of $B_1$, discussed above.

TABLE I.—*Initial Thyroid Status and Survival*

| $B_1$ | Total | Duration of life (years) 0 | 1 | 2 | 3 | 4 | 5+ | 5-Year survival rate |
|---|---|---|---|---|---|---|---|---|
| < 10 | 1 | — | 1 | — | — | — | — | — |
| 10–19 | 5 | 1 | 1 | — | — | — | 3 | 60·0% |
| 20–29 | 21 | — | 3 | 3 | 4 | 1 | 10 | 48·0% |
| 30–39 | 33 | 1 | 10 | 3 | — | 1 | 18 | 54·0% |
| | 60 | 2 | 15 | 6 | 4 | 2 | 31 | 51·7% |
| 40–49 | 37 | 2 | 8 | 2 | — | 3 | 22 | 59·0% |
| 50–59 | 16 | — | 2 | 5 | — | 1 | 8 | 50·0% |
| 60–69 | 6 | 2 | — | — | — | — | 4 | 67·0% |
| | 59 | 4 | 10 | 7 | — | 4 | 34 | 57·6% |

Although the 5-year survival rate has been calculated for each group of $B_1$ values, the numbers are small in such a classification and there is no statistically significant difference between the rates, nor is any trend discernible. For the division of the series at the value of 40 for $B_1$, Fig. 4 shows the survival rates by year up to the 5th. Again there is no real difference between the curves. The fact that both these curves are atypical for survival experience in the 1st year in particular reflects merely an initial selection which, as we have pointed out earlier, excluded advanced cases and those too ill for $^{131}I$ investigations to be made. Further evidence of the deliberate selection by stage of advancement is shown in Table II, which sets out in a similar way for grouped values of $B_1$ the stage distribution of cases in the series. The mean stage of the first group ($B_1$ less than 40) is 1·97, with a 5-year survival rate of 51·7%, and for the second group mean stage is 2·03, and 5-year survival rate 57·6%. Neither difference is statistically significant.

We have classified according to condition at death the 69 patients who died within 5 years from the time their $B_1$ value was obtained. Of 50 cases with distant or widespread metastases, the mean $B_1$ figure was 38·34; of 14 cases with only local metastases it was 38·2; and for the remaining 5 cases with no evidence of malignancy it was 41·8. Fifty cases survived the 5 years, but 3 refused the $B_2$ determination. Of the other 47 (mean $B_1$ = 38·68, mean $B_2$ = 34·68) 38 showed no evidence of malignancy, but 9 had developed secondaries (mean $B_1$ = 42·3, mean $B_2$ = 35·8). Again, none of these differences is statistically significant.

We are forced to the conclusion, therefore, from the results in our series of 119 cases that the prognosis in breast cancer seems not to be influenced by the

FIG. 4.—Comparison of survival rates of patients having initial thyroid activity below median ($B_1 < 40$) with those at median and above ($B_1 = 40+$).

TABLE II.—*Initial Thyroid Status and Stage Distribution*

| $B_1$ | Stage 1 | 2 | 3 | 4 | Total | |
|---|---|---|---|---|---|---|
| < 10 | 1 | — | — | — | 1 | Mean stage |
| 10–19 | 4 | 1 | — | — | 5 | = 1·97 |
| 20–29 | 4 | 11 | 5 | 1 | 21 | 5-Year survival |
| 30–39 | 11 | 12 | 9 | 1 | 33 | rate |
| | 20 | 24 | 14 | 2 | 60 | = 51·7% |
| 40–49 | 12 | 11 | 10 | 4 | 37 | Mean stage |
| 50–59 | 8 | 5 | 2 | 1 | 16 | = 2·03 |
| 60–69 | 2 | 3 | — | 1 | 6 | 5-Year survival rate |
| | 22 | 19 | 12 | 6 | 59 | = 57·6% |

level of thyroid activity determined at the outset by the method we have described. We feel that we cannot however regard the matter as conclusively settled on the basis of these results. It may well be that our measure of activity is not the best to use, nor made at the optimum time. Serial measurements, perhaps 6-monthly, would be likely to yield more valuable information, but could only be entertained if, like the estimation of P.B.I., they caused a minimum of inconvenience to patients.

### DISCUSSION

The fact that our own results, like so many others in the literature, are inconclusive or negative is puzzling in view of the strength of clinical impressions to the contrary. Reeve *et al.* (1961) suggested a possible explanation of the conflict between their own findings and those of Edelstyn *et al.* (1958), which they had confirmed by re-analysis, which implied that among patients with disseminated disease there might be a degree of disorganisation in the various measures of thyroid activity that have been used. We have ourselves suspected that we might not be using the best indices of thyroid function, and we were interested to recieve some support for this view in a communication from Reynolds (1965, personal communication). Reynolds is attempting to assess the potential functional activity of the thyroid gland by its response to exogenous stimulation, by determining the $^{131}I$ uptake first before, and then after, the administration of T.S.H. A normal gland is likely to show a sharp increase in uptake, whereas one that is already maximally stimulated will not. Whether or not this method will effect a resolution of the apparent anomalies so far reported can only be decided by trial, but we hope to be able to extend our own studies in this direction.

### SUMMARY

The results of an investigation to assess the influence of thyroid activity on prognosis in breast cancer are reported. Two measures of thyroid activity have been used, the uptake at the 24th hour (" B ") of tracer doses of $^{131}I$ and the assay of protein-bound iodine (P.B.I.), which are shown to be closely equivalent in practice. Patients who survived 5 years from an initial measurement of B were assessed in the same way again, and showed a slight reduction, not statistically significant, in thyroid activity. In relation to their initial measurements the actual fate of patients 5 years later appeared to show no evidence of the influence of thyroid activity. This apparently anomalous conclusion, conforming with others in the literature, is attributed to the possible use of an inappropriate measure of activity. It is therefore proposed to attempt a measurement of the gland's *potential* activity by its response to exogenous stimulation.

We wish to thank Mr. J. D. F. Williams, principal physicist at the Coventry and Warwickshire Hospital, for undertaking the iodine studies and Miss Molly Alsop for much secretarial and computational assistance.

### REFERENCES

Capelli, L. and Margottini, M.—(1964) *Acta Un. int. Cancr.*, **20**, 1493.
Edelstyn, G. A., Lyons, A. R. and Welbourn, R. B.—(1958) *Lancet*, i, 670.
Emery, E. W. and Trotter, W. R.—(1963) *Lancet*, i, 358.
Humphrey, L. J. and Swerdlow, M.—(1964) *Cancer, N.Y.*, **17**, 1170.
Lyons, A. R. and Edelstyn, G. A.—(1965) *Br. J. Cancer*, **19**, 116.
Reeve, T. S., Hales, I. B., Rundle, F. F., Myhill, J. and Croydon, M.—(1961) *Lancet*, i, 632.
Sicher, K. and Waterhouse, J. A. H.—(1961) *Br. J. Cancer*, **15**, 45.
Stoll, B. A., (1962) *Br. J. Cancer*, **16**, 436.